# Analysis of MAP kinase MPK4/MEKK1/MKK genes of Carica papaya L. comparative to other plant homologues

**DOI:** 10.6026/97320630013031

**Published:** 2017-02-28

**Authors:** Muhammad Hanam Hamid, Lina Rozano, Wee Chien Yeong, Janna Ong Abdullah, Noor Baity Saidi

**Affiliations:** 1 Biotechnology and Nanotechnology Research Centre, Malaysian Agricultural Research and Development Institute, 43400 Serdang, Selangor, Malaysia;; 2 Department of Cell and Molecular Biology, Faculty of Biotechnology and Biomolecular Science, Universiti Putra Malaysia, 43400 UPM Serdang, Selangor, Malaysia;

**Keywords:** MPK4, MEKK1, MKK1, MKK2, MAP Kinase Cascade, Papaya

## Abstract

Mitogen-activated protein kinase 4 (MPK4) interacts with the (Mitogen-activated protein kinase kinase kinase 1) MEKK1/ Mitogenactivated protein kinase kinase 1 (MKK1)/ Mitogen-activated protein kinase kinase 2 (MKK2) complex to affect its function in plant development or against pathogen attacks. The KEGG (Kyoto Encyclopedia of Genes and Genomes) network analysis of Arabidopsis thaliana revealed close interactions between those four genes in the same plant-pathogen interaction pathway, which warrants further study of these genes due to their evolutionary conservation in different plant species. Through targeting the signature sequence in MPK4 of papaya using orthologs from Arabidopsis, the predicted sequence of MPK4 was studied using a comparative in silico approach between different plant species and the MAP cascade complex of MEKK1/MKK1/MKK2. This paper reported that MPK4 was highly conserved in papaya with 93% identical across more than 500 bases compared in each species predicted. Slight variations found in the MEKK1/MKK1/MKK2 complex nevertheless still illustrated sequence similarities between most of the species. Localization of each gene in the cascade network was also predicted, potentiating future functional verification of these genes interactions using knock out or/and gene silencing tactics.

## Background

Defense mechanisms in plants towards pathogens infection lay in a complicated web of multiple genes interactions in the defense response pathways. Mitogen-activated protein kinase (MAP kinase) cascades are critically involved in regulating plant defense mechanisms including plant innate immune responses [1]. Studies on the MAP kinase cascade in plants had led to the discovery of groups of genes interacting with each other to activate or inhibit defense related genes such as in pathogenesis related (PR) genes [2]. This mitogen-activated protein kinase 4 (MPK4) is a nuclear and cytoplasmic localized protein involved in mediating responses to pathogen [3].

Study of the MPK4/MPK4-like gene is common in the model plant, Arabidopsis. However, there is still lack of information in non-model plant species including papaya. Interestingly in papaya, MPK4 was found to magnify the defense responses against papaya dieback disease caused by Erwinia mallotivora [4] and the papaya crown rot disease by Erwinia papayae. These two common pathogens showed symptoms that affect vascular tissues especially towards the upper merismatic stem of the papaya tree [4-5]. Based on the TAIR database [6], MPK4 was highly expressed throughout the entire plant including the vascular bundles, which served as the colonization points for those pathogens [7]. Thus, it is essential to strategize the MPK4 role in systemic acquired resistances (SARs) and strengthen the plant’s sentry throughout the vascular bundles.

The unique and complex interaction of MPK4 within the MAP kinase cascade also warrants a crucial in silico study of the MPK4 gene concomitantly with other genes in the cascade that interact directly with it. Activation and regulation of MPK4 in the MAP kinase cascade were noted to require MEKK1 for cell death defense response in Arabidopsis [8] and MKK1/MKK2 complex in the defense related pathways. MPK4 of group B in the MAP kinase cascade associates with MEKK1 and interacts with MKK1 and MKK2 to suppress the activation of innate immune response in plants [2] [9-11]. MPK4 is also stimulated by flg22, and is involved in flagellin and reactive oxygen species (ROS) signalings [12-14]. Considering MEKK1, MKK1/MKK2 and MPK4 are closely associated to form a kinase cascade; interaction study of each gene across different plant species would be valuable.

In this study, the MPK4 gene from papaya was extracted based on Arabidopsis MPK4 (AtMPK4) orthologs and the sequence was analysed using bioinformatics approach to predict its characterized functions and interactions in the MAP kinase cascade in papaya in comparison to other plants. The MEKK1, MKK1 and MKK2 orthologs were also mined from the Arabidopsis genes designated as AtMEKK1, AtMKK1 and AtMKK2, respectively. Homologs of MPK4 in soybean showed MPK4 functioned to negatively regulate defense responses but positively control growth and development [15]. Another study also revealed the homologs interaction of MPK4 and its neighbouring genes in the MAP kinase cascade had adverse regulation on other members of the MAP kinase protein family [16]. Thus, in silico study of MPK4 and its neighbouring members in the papaya MAP kinase cascade, covering the structural proposition and signature similarities between papaya and other plants known for plant-pathogen interaction mapping based on the KEGG pathways, is necessary to facilitate further insights into predicting MPK4 function and its interaction within the MAP kinase cascade particularly in papaya responses against pathogen.

## Methodology

### Data collection

Sequences of AtMPK4, AtMEKK1, AtMKK1 and AtMKK2 were obtained from the Arabidopsis Information Resource (TAIR) database [17] and orthologs from the Arabidopsis were blasted using Phytozome database version 10.3 [18] for each selected plants specifically papaya (Carica papaya), Japanese rice (Oryza sativa Japonica L), cucumber (Cucumis sativus), corn (Zea mays), tomato (Solanum lycopersicum), grape (Vitis vinifera), sorghum (Sorghum bicolor) and soybean (Glycine max). The sequences acquired were the coding sequences (CDS) with the highest hit scores and smallest E-values in the database excluding MEKK1 sequence from the Vitis vinifera genome database and MKK2 sequence from the Oryza sativa genome database, which showed no blast matches.

### In silico sequence analysis

The base content percentage of each sequence was calculated using the GC Calculator from BiologicsCorps [19]. The nucleotide sequences were analyzed and translated into amino acid sequences using the Mega software version 6 [20] and matched with predicted protein sequences from the Phytozome database [18]. The mined data showed that the reference genes, AtMKK1 and AtMKK2, had the lowest E-values hits as the MKK1/MKK2 genes from the Carica papaya genome database of Phytozome. The sequence, evm.model.supercontig_371.4, of Carica papaya showed high similarity to both AtMKK1 and AtMKK2. Thus this sequence was used throughout the analyses of MKK1 and MKK2 of papaya.

Translated nucleotides sequences that matched with the database were assessed using the ScanProsite software [21] to predict signature motifs and domains shared by each plant species [22]. Upon identification, localization of the structural features in each domain and motif were determined and compared to each plant species. Multiple sequences alignment of the protein sequences was created using the t-coffee software established by the EMBL European Bioinformatics Institute (EBI) [23] and viewed with the BioEdit software version 7.2.5 [24]. The t-coffee software was also used to analyse the matrix homology identity of each sequence in order to study the homology percentage shared between the plant species [25].

The evolutionary path of each gene in different plant species was evaluated using the neighbour-joining phylogenetic trees constructed using the Mega software version 6 [20] with bootstrap values of 500 replicates. The substitution model was carried out using the p-distance method while pairwise deletion was opted to treat gaps or missing data within the compared sequences.

Further study was carried out using the Pathway Studio software web version [26] to predict localization and interaction of corresponding genes involved in defense responses including defense against pathogens. Mapping of the protein-protein interaction was based on the IntAct and MINT databases followed by visualization in the Cytoscape software version 3.1 [27], which was integrated with the text mining information from the Agilent literature search plugins. Interaction of the MPK4- MEKK1-MKK1/MKK2 genes was studied based on orthologous gene information from the Arabidopsis MAP kinase cascade for each respective genes.

The papaya MPK4, MEKK1 and MKK1/MKK2 sequences were blasted into Blast2GO version 3.2 [28] for the genes’ ontologies (GO) and respective functions regarding biological process, molecular functions and cellular component [29-30].

## Result and discussion

### Sequence variations and base contents

Sequences were obtained from the Phytozome database for each gene of each available plant, respectively, with nine sequences for MPK4, eight for MEKK1, nine for MKK1 and eight for MKK2. There were no available sequences of MEKK1 in grape and MKK2 in Japanese rice. The length of the MPK4 sequence from each plant shows a slight difference from each other, approximately 1100 base pairs (bp). The longest sequence of MPK4 was from sorghum with 1167bp while the shortest sequence was from cucumber with 1113bp. The MEKK1 sequences, however, were dissimilar in nucleotides sequence length ranging from 663bp (Japanese rice) to 1998bp (tomato). While most of the sequences were approximately 2000bp in length, only Japanese rice (663bp) were shorter by 1000bp. The MKK1 sequences were relatively similar to each other with 50 to 74bp larger than 1 kilo base pair (kb) length. In contrast, the MKK1 sequence from soybean was only 807bp. The longest sequence of MKK1 obtained was from tomato with 1074bp. The longest sequence of MKK2 mined was from the Arabidopsis genome with 1119bp while the shortest sequence was in sorghum with only 69bp differences. The least variation in sequence length between the species corresponded to higher chance of conserved region within the particular gene. In the case of verification of the MPK4 gene in papaya, we concluded that the MPK4 sequence obtained was closely related to the AtMPK4, likewise maybe its function too. Whereas in MEKK1 and MKK1, high variations of length sequences were acquired in one to two species which was out of the comparative range (±100bp).

**Figure 1:**
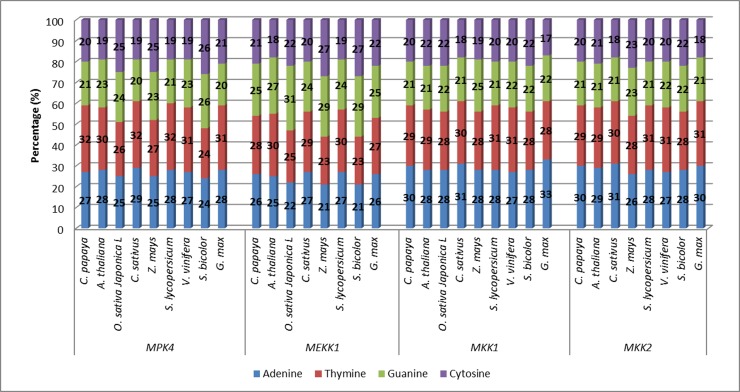
Comparison of bases content for MPK4, MEKK1, MKK1 and MKK2 genes in different plant species is shown.

Comparing the base content analysis of each MPK4, MEKK1, MKK1 and MKK2 sequences, the lowest percentage of individual base was cytosine while the highest was adenine, both in MKK1 of soybean (Figure 1). GmMKK1 gene sequence was the shortest in length for the gene represented as compared to other species. The maximum percentage of MKK1’s GC-content was equivalent in the Japanese rice, corn and sorghum. Cucumber and soybean GC-content in MKK2 also scored the lowest percentage (39%) while the corn marked the uppermost value with 46%. One of the key parameters of genome sequence variations in evolutionary study reported was the genomic GC-content, which was confined to between 25% and 75% [31]. Relatedness of both CDS length and GC-content percentage was discussed by Oliver & Marin (1996) [32]. The CDS length was shown to be under both functional and structural constraints [33-36]. The CDS length refers to the size distributions of the gene parts (exons, introns, leader and trailer regions, etc.) and is known to be under a stabilizing selection pressure against extreme lengths [37]. These points support the conclusion that the GC percentage effects on the CDS length might constitute a new evolutionary meaning for compositional variations in DNA GC-content.

### Variation of stop codon

**Figure 2:**
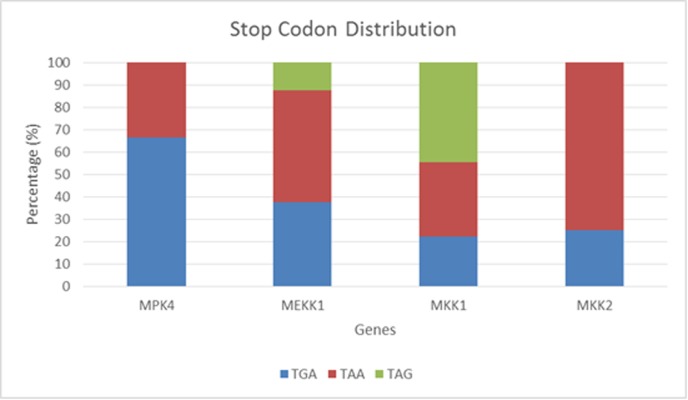
Percentage distribution of stop codon variation between genes. Stop codon variation occur more often in MEKK1 and MKK1 genes compared to MPK4 and MKK2 genes, with TAG present in both MEKK1 and MKK1 genes.

Diversity of the three types of stop codon in the CDS sequences was observed in MEKK1 and MKK1 sequences, while MPK4 and MKK2 sequences revealed only diversity in TGA and TAA. Only Japanese rice, corn and sorghum have TAA in the MPK4 sequences while most other species consist of TGA. In the MKK2 sequence, TAA is commonly found whereas only papaya and sorghum contain TGA. Almost half of the species have TAG in the MKK1 gene while the remaining has distribution of 33.33% TAA and 22.22% TGA. Stop codons in MEKK1 are distributed with only one TAG stop codon (sorghum), and respectively three and four stop codons of TGA and TAA between plant species. The percentage of stop codon variations was diverged between genes as represented (Figure 2). From the percentage of distribution in all species, TAA is more common as a stop codon in every sequence especially in MKK2 gene while TAG appears the least favourable.

### Signature motifs and domains

Analyses of peptide sequences from the translated genes of each species via domain and motif predictions using the ScanProsite software revealed similar domain of protein kinase. The features studied from the conserved domains and motifs in the MPK4, MEKK1, MKK1 and MKK2 for each plant species.

Thirty out of 35 sequences studied shared similar motif signature region of the protein kinase ATP-binding and serine/threonine protein kinase active-site signatures. The domain region in all the MPK4 from each species demonstrated the same 286bp length. These MPK4 sequences had the conserved MAP kinase signature region except in sorghum, which exhibited a glycine rich region profile and lipid cysteine motifs of S-diacylglycerol and Npalmitoyl. The MPK4 of sorghum contained tyrosine protein kinase specific active site signature at positions 177-189bp. In contrast, no MAP kinase signature was obtained from the other three genes sequences. These genes share the protein kinase domain with minor distinction of locations and lengths of binding domain ranging from 117 to 286bp. Interestingly, MKK1 and MKK2 in cotton were found with the presence of TonBdependent receptor protein signature but lacking the kinase ATPbinding site.

Study of the predicted domains and motifs indicate variance in length of the protein kinase ATP-binding site in different genes in which MPK4 had the longest region of 25bp, MEKK1 with the shortest region of 23bp while MKK1/MKK2 with 24bp. The prediction analysis also found the absence of the proton acceptoractive site in the MEKK1 sequence. Amino acids changes were noted in D482F in the papaya MEKK1 protein sequence, D220S in the Japanese rice MEKK1 protein sequence and K159R soybean MKK1 protein sequence. These changes could be due to evolutionary mutation processes.

### Multiple peptide sequences alignment and gene sequences identity matrix homology

**Figure 3:**
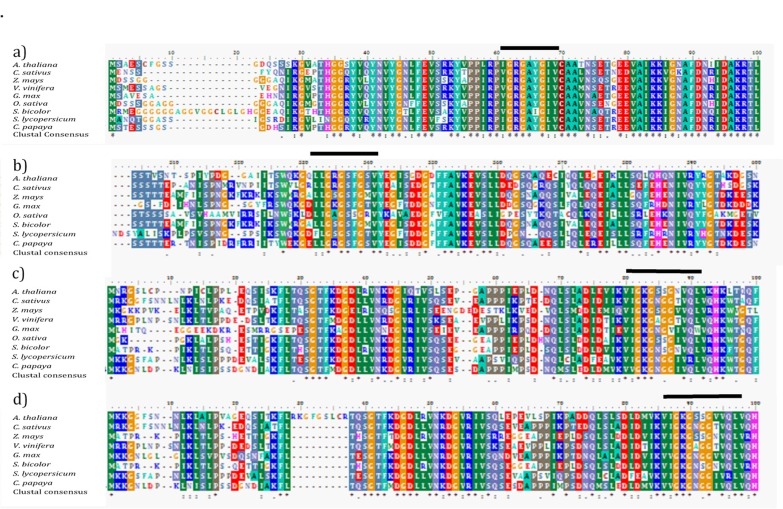
Multiple peptide sequences alignment of MAP kinase proteins from different plant species. Conserved protein kinase domain regions shared by each sequences are as represented with black line above the region. Sequences alignment of (a) MPK4 genes, (b) MEKK1 genes, (c) MKK1 genes, and (d) MKK2 genes.

Figure 3 summarises the multiple sequences alignment for each peptides using the t-coffee software, which depicted the conserved regions between the plants species. The conserved domain of the protein kinase attained from the ScanProsite was marked with a black line above the sequences alignment, specific for each region. This protein kinase region was conserved in each orthologs especially for MPK4, which only differed in three peptides out of ten conserved peptides in the domain. However, this kinase domain was the least conserved in the MKK1 sequence alignment having only five similar peptides shared between the plant species across 12 peptides.

The identity matrix of each genes sequence alignment calculated using the t-coffee software discovered the papaya MPK4 gene had the highest identical value (92.25%) with grape MPK4 gene while the papaya MEKK1 gene had the highest identical value (59.02%) with cucumber MEKK1 gene. The papaya MKK1 and MKK2 also shared high sequence homology with the grape at 77.90% and 78.19%, respectively. Although MEKK1 in grape was undiscovered, other three genes studied in papaya showed closest homology in term of sequence composition to the sequences in grape with 77% to 92%.

### Neighbour-joining phylogenetic trees

The significant differences observed between each MPK4 sequences across the plant species are postulated as a response to evolutionary pressure imposed on the gene to maintain its core function as a defense gene against certain host-specific pathogens. For each respective variation in the sequences throughout the plant species, they were grouped into two major clusters consisting of dicotyledonous plants (Class: Magnoliopsida) versus monocotyledonous plants (Class: Liliopsida).

**Figure 4:**
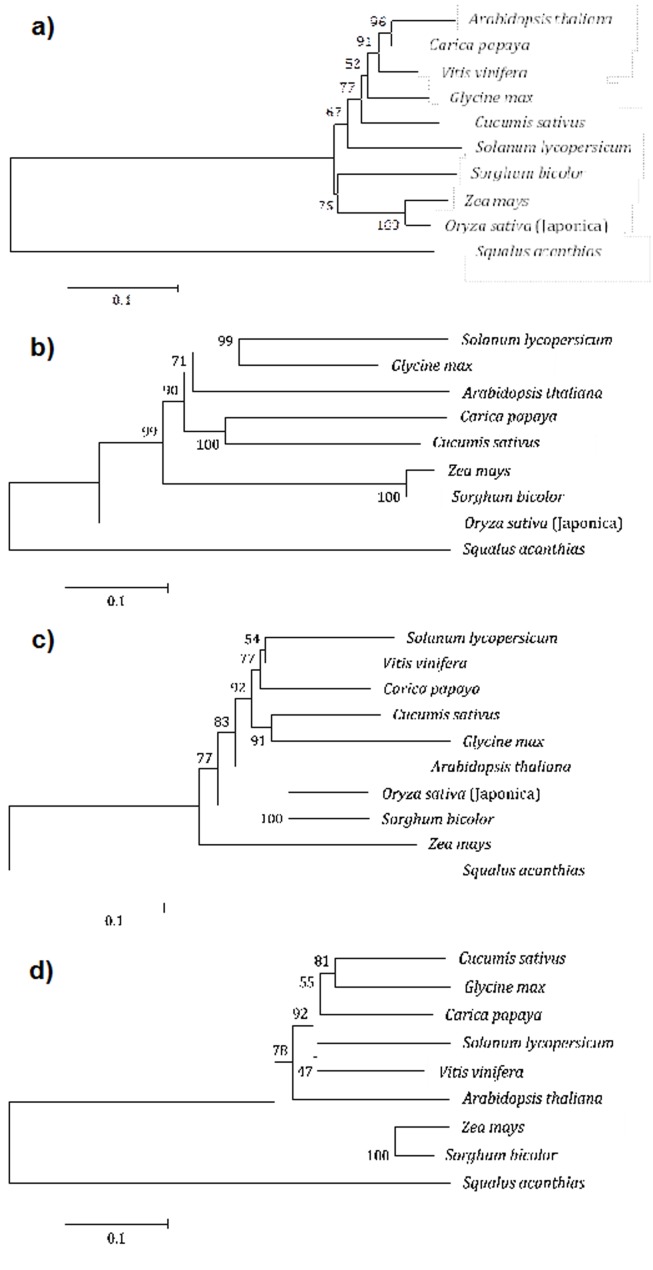
Neighbour-joining phylogenetic tree of MAP kinase genes in different plant species with an out-group of spiny dogfish, Squalus acanthias (gi|55724220 |gb|CV798545.1). The scale bar corresponds to 0.1 substitutions per site, with bootstrap values of 500 replicates. Phylogenetic tree of (a) MPK4 genes with 25 to 100 bootstrap value per generated nodes, (b) MEKK1 genes with 71 to 100 bootstrap value, (c) MKK1 genes with 54 to 100 bootstrap value, and (d) MKK2 genes with 47 to 100 bootstrap value.

The evolution of the MPK4 gene between the plant species was distinctive in those two clusters. The papaya MPK4 was clustered under the same clade with Arabidopsis (96%), while tomato MPK4 (67%) showed a distant lineage from those groups as illustrated in (Figure 4a). Within the monocotyledonous group, corn and Japonica rice were narrowly similar to each other (100%) compared to sorghum (25%). The orthologs study using the Arabidopsis sequence as a reference to predict the papaya MPK4 sequence revealed close relatedness between them. This, however, contradicted the displayed result showing closer relatedness of most of the species to the rice and corn.

The phylogenetic analysis of MEKK1 showed high (71 to 100%) bootstrap support across the species (Figure 4b). These values present a high confidence of accuracy that the clades were defined by the nodes in MEKK1. With the exception of rice, most of the MEKK1 genes were highly conserved between the species. Although their protein features analysis was absent in proton acceptor as in rice, the close relationship of papaya MEKK1 sequence to the cucumber’s suggests a high possibility of similar domain functions but a lack of residues required for phosphotranferase activity. The lack of certain amino acids or sites in a domain, which is typically conserved in the catalytic loop, does not necessarily imply its non-functionality like its comparative reference such as in Arabidopsis, but might be due to the occurrence of mutation based on plant genetic variation. This condition was termed as a pseudokinase domain.

Further phylogeny analysis for MKK1 suggested that the sequence was closely associated between rice’s and sorghum’s (Figure 4c). In contrast, cucumber and soybean genes had a closer relationship for MKK2 (Figure 4d) sequences compared to tomato’s and grape’s. This finding contradicted the homology data of identity matrix between MKK1/MKK2 for papaya sequence.

To sum the sequence analysis of corresponding genes, MPK4 scored high in conservation and stability across the plant species. It had less variance of stop codons and GC content, contained specific domain features, high conservation and sequence homology identities, and close distance of phylogeny. The MEKK1, however, lack domain features, and has low (below 60% homology) sequence identity. Nevertheless, its high bootstrap of more than 70% confidence is likely true across the cluster group between the species analysed. Both the MKK1 and MKK2 lacked certain features in some species but were conserved in papaya, with higher homology identity index for MKK2 disputed to MKK1, which had a closer distance referred to papaya sequence.

### Gene-gene interaction in defense response

**Figure 5:**
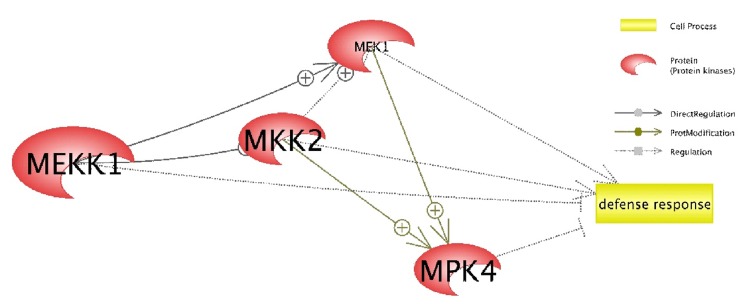
Schematic diagram of gene-gene interaction network of MPK4, MEKK1, MKK1 (labelled as MEK1) and MKK2 in plant defense response. “+” symbol associated with interaction arrows indicate the activation of gene corresponding to the upstream gene.

Searches using the Pathway Studio software [26] successfully identified potential interactions between genes that modulated the defense responses) as shown in (Figure 5). Within the MAP kinase cascade involving MPK4, MEKK1, MKK1 (also named as MEK1) and MKK2, the activation of direct regulation, protein modification and regulation processes were also identified.

Based on in silico study of MPK4 and MEKK1/MKK1/MKK2 complex interaction using the Pathway Studio software, it was reported that all four related genes were involved in the regulation of defense response. In a study of the genetic manipulation of MAP kinase cascade in plants proposed that the MPK4, MEKK1, and MKK1/MKK2 complex negatively regulated defense responses [38]. Based on this result, we predict that papaya MPK4 and MEKK1 also regulate defense response by suppressing it once it is activated.

Within the MAP kinase cascade, it was highlighted that the multiple levels of signaling pathway starting from the MAP kinase kinase kinase (MAPKKK) to the MAP kinase kinase (MAPKK), and then to the activation of the MAP kinase (MAPK), leading to signal plant defense modulation [39]. This pathway model was later reviewed by Bethke et al. (2009) who studied the signaling pathway between the MAP kinase and ethylene signalling [40]. A mitogen-receptor-MAPKKK-MAPKK-MAPKtarget- cellular response of a MAPK module was presented in a plant–pathogen interaction. Based on the close comparison of the above mentioned pathway model to the Pathway Studio, we hypothesized that the signaling ensued from the MEKK1 to MKK1/MKK2 complex would lead to subsequent activation of the MPK4, to regulate plant defense response in papaya (Figure 5).

Upon infection, the MEKK1 received signal from the receptor and triggered the MKK1/MKK2 complex with consequent activation of MPK4 [2] [41-42]. The role of MEKK1 to suppress defense response had been as the loss of the MEKK1 mutant resulted in the activation of a defense response [8]. MEKK1 was advocated to induce defense response such as in the production of reactive oxygen species, and the induction of a hypersensitive response and localized cell death at the site of infection [43].

We postulated that signals from MEKK1 will lead to the expression of MKK1 and MKK2 as these two complexes promote protein modification interaction to stimulate MPK4 expression. MKK1/MKK2 functions as an important intermediate, which interacts between MEKK1 and MPK4. Within Arabidopsis, MKK1/MKK2 was conveyed to have overlapping functions in defense signaling mediated by MEKK1 and MPK4 [44]. It can be concluded that both MKK1 and MKK2 interact in similar regulation towards MPK4 activation, once both MKK1 and MKK2 have been induced by MEKK1. Thus, we propose that the cooperative signaling of MKK1/MKK2 as a complex play an important role in promoting defense response. Co-dependency of MKK1/MKK2 interactions was tested using Zea mays kinase cascade in transgenic tobacco where MEKK1, induced by H2O2, was proven to activate this complex and subsequently led to MPK4 expression [45].

Inactive MPK4 was described to be phosphorylated by MKK1 at the threonine site of MPK4 to trigger defense response [46]. Phosphorylation of MPK4 was also reported to be activated by MKK2 [47] to promote defense response, while it was also stated that the phosphorylation was induced by cold and salt stresses [48]. Modification of MPK4 by MKK1/MKK2 complex was later detailed by Liang et al., (2013) where the phosphorylation was defined, caused by upstream induction of MKK1/MKK2 and MEKK1 [49]. Once phosphorylated, MPK4 triggers subsequent response for antimicrobial production. The role of the cascade activity thru a specific phosphorylation pathway between MEKK1-MKK1/MKK2 and MPK4 for defense and stress response was also reported in Arabidopsis [50] and in rice [51].

The interaction of MPK4 in the MAPK cascade together with the upstream components, MKK1/MKK2 and MEKK1, was found to be involved in the phosphorylation pathway as a negative regulator of systemic-acquired resistance (SAR) [8]. While this interaction was reported to be mainly involved in stress responses, specific communication between MPK4 and its MAP kinase cascade upstream was predicted to be activated by a diverse set of environmental responses including pathogens attack, cold stress, osmotic stress and oxidative stress [52].

**Figure 6:**
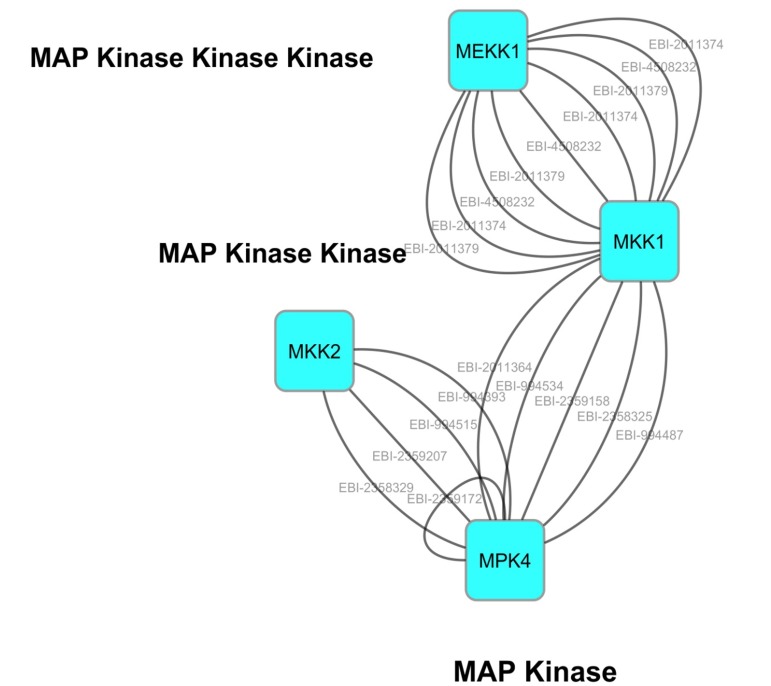
MAPK kinase cascade depicting MPK4, MEKK1, MKK1 and MKK2 interactions in plant defense response. This network was built using the Cytoscape databases, to link candidate genes to each other. Interactions between genes are proved to support coexpression associated especially MEKK1 gene towards MKK1, and subsequently towards MPK4. The studied interactions between genes are referred with the EBI (European Bioinformatics Institute) identification numbers.

Analysis of the MPK4 cascade via the Cytoscape software (Figure 6) revealed significant interaction of MEKK1-MKK1-MKK2- MPK4 within the cascade. MKK1 plays an imperative role in the interaction between MEKK1 and MPK4; however no evidence of direct interaction between MEKK1 and MKK1 with MKK2 has been reported. In line with our postulation of the cooperative interaction between papaya MKK1 and MKK2 to phosphorylate MPK4, we propose that MEKK1 would only interact via MKK1 and not MKK2 nor directly to MPK4. While MKK2, presented by the Pathway Studio, demonstrated significant interactions between MEKK1 and MKK1 with MKK2, we surmise the acts of potential intermediate genes to work between MEKK1 signaling to MKK2 in order to play out its functions. The potential role of MPK11 was suggested in the interaction between closely related MAPKKs of MKK1 and MKK2 in Arabidopsis [53]. Supplementary study on MPK11 related to MKK1 and MKK2 resulted in a phosphorylation reaction type of interaction between MKK1-MPK11 (EBI-2359178) and MKK2-MPK11 (EBI- 2358753). Additional physical association interaction was also detected corresponding to MKK1-MPK11 interaction [53].

The detailed studied interactions between genes extracted from the Cytoscape database whereby two different interaction types were performed to study the genes interaction. The two hybrid and two hybrid array detection methods related a physical association type of interaction, while the protein kinase assay and in-gel kinase assay showed a phosphorylation reaction type of interaction. The overall data shown supports the interaction of MEKK1-MKK1/MKK2-MPK4 as proposed in Figure 6.

Additional analysis performed using the Blast2GO software with MEKK1, MKK1/MKK2 and MPK4 sequences in papaya to study the potential functions as indicated in the gene ontology data showed a transferring phosphorus-containing group of enzyme (Enzyme code (EC): 2.7.11) with 20 hits and 0.0e0 e-value. The attained gene ontologies of each sequence are summarised. CpMEKK1 showed the highest possibility of classes in signal transduction and cellular protein modification (biological process aspect); ion binding and kinase activity (molecular function aspect); and intracellular related function (cellular components aspect). CpMPK4 also portrayed five possible gene ontology classes of functions including cellular protein modification process (biological process); ion binding, signal transducer activity and kinase activity (molecular function); and intracellular related function (cell components aspect). Both of the postulated CpMKK1/MKK2 from the single sequence showed a group of eight classes of functions: cell death, signal transduction, cellular protein modification process, cell cycle and response to stress (biological process); ion binding and kinase activity (molecular functions); and cytoplasm related function (cellular component).

As gene ontology study of MEKK1, MKK1/MKK2 and MPK4 in papaya emanated mainly in signal transduction, ion binding and kinase activity, interaction between each gene seemed essential especially in the MAP kinase cascade studied. Biological process of response to stress discovered in CpMKK1/MKK2 may demonstrate more direct approach of the gene to play its function against stress. This result might also hypothesise the lesser interaction as proposed in MKK2 interaction of the Cytoscape result (Figure 6). The similar sequence of both CpMKK1 and CpMKK2 obtained leads us to acquiesce that CpMKK1/MKK2 has a similar function of MKK1 and MKK2 whether to act based on signalling from MEKK1 or direct signalling from stress induction.

Considering the MEKK1-MKK1/MKK2-MPK4 module was expected to negatively regulate the defense response in papaya, a good suggestion put forth by Bigeard et al. (2015) that a mutant of this cascade be created using knock-out gene approach to represent a constitutive defense responses including a constitutive expression of the pathogenesis-related (PR) defense genes, would indeed elucidate the intricacies of the players involved in conferring resistance of plants against potential pathogens [54]. Here, our findings spotlight the possible complex interaction of MEKK1-MKK1/MKK2-MPK4 in the papaya MAP kinase cascade as a focal point of a busy genetic highway between pathogen–plant defense signaling pathways culminating in either a susceptible or resistant response worth further in depth study.

## Conclusion

The analysis of the conserved regions and signature motifs of MPK4 and MEKK1/MKK1/MKK2 complex genes support the role each gene plays in the MAP kinase cascade in promoting defense response. Overall, MPK4 indicates high conservation and stability in papaya and other plant species investigated. The protein kinase domain and motifs of MPK4 justified with high estimates of the conservation, homology and sequence propositions across the plant species analysed. The findings from this in silico study revealed that the conservation of the candidate genes analysed particularly MPK4 within each plant species are functionally reliable as compared to the referenced sequences validated through knock out gene study of MPK4 in Arabidopsis. The in silico study of the MPK4 gene in the MAP kinase cascade and the MEKK1/MKK1/MKK2 complex showed wide variations in stop codons, GC-content and homology identity but yet conserved in their respective signature domains to assume their basic functional role, and thus to make use of these interactions behaviour between genes isolated for future references.
